# Abnormal Fixational Eye Movements in Amblyopia

**DOI:** 10.1371/journal.pone.0149953

**Published:** 2016-03-01

**Authors:** Aasef G. Shaikh, Jorge Otero-Millan, Priyanka Kumar, Fatema F. Ghasia

**Affiliations:** 1 Daroff-Del’Osso Ocular Motility Laboratory, Louis Stokes Cleveland VA Medical Center, Cleveland, Ohio, United States of America; 2 Department of Neurology, Case Western Reserve University, Cleveland, Ohio, United States of America; 3 Neurology Service, Louis Stokes Cleveland VA Medical Center, Cleveland, Ohio, United States of America; 4 Department of Neurology, Johns Hopkins School of Medicine, Baltimore, Maryland, United States of America; 5 Cole Eye Institute, Cleveland Clinic, Cleveland, Ohio, United States of America; State University of New York Downstate Medical Center, UNITED STATES

## Abstract

**Purpose:**

Fixational saccades shift the foveal image to counteract visual fading related to neural adaptation. Drifts are slow eye movements between two adjacent fixational saccades. We quantified fixational saccades and asked whether their changes could be attributed to pathologic drifts seen in amblyopia, one of the most common causes of blindness in childhood.

**Methods:**

Thirty-six pediatric subjects with varying severity of amblyopia and eleven healthy age-matched controls held their gaze on a visual target. Eye movements were measured with high-resolution video-oculography during fellow eye-viewing and amblyopic eye-viewing conditions. Fixational saccades and drifts were analyzed in the amblyopic and fellow eye and compared with controls.

**Results:**

We found an increase in the amplitude with decreased frequency of fixational saccades in children with amblyopia. These alterations in fixational eye movements correlated with the severity of their amblyopia. There was also an increase in eye position variance during drifts in amblyopes. There was no correlation between the eye position variance or the eye velocity during ocular drifts and the amplitude of subsequent fixational saccade. Our findings suggest that abnormalities in fixational saccades in amblyopia are independent of the ocular drift.

**Discussion:**

This investigation of amblyopia in pediatric age group quantitatively characterizes the fixation instability. Impaired properties of fixational saccades could be the consequence of abnormal processing and reorganization of the visual system in amblyopia. Paucity in the visual feedback during amblyopic eye-viewing condition can attribute to the increased eye position variance and drift velocity.

## Introduction

Amblyopia refers to a reduction of best-corrected visual acuity that cannot be attributed to structural eye abnormalities[1,2]. Amblyopia is caused by abnormal visual experience in early childhood leading to functional and anatomic abnormalities in cortical area V1[3,4]. Such alterations in the area V1 account for various sensory deficits including diminished visual acuity, contrast sensitivity, spatial and temporal crowding and abnormal binocularity [5–7]. Visual acuity depends on where the image falls on the retina with the highest resolution achieved in the foveola.

Although stable fixation is a prerequisite for clear vision, constant stimulation at a single foveal location can progressively weaken the neural responses leading to visual fading. Involuntary physiological eye movement cause subtle changes in the foveal position of the target image and counteracts fading[8–14]. Such physiological involuntary movements are microsaccades, drifts, and ocular tremor. Microsaccade related change in the single unit activity in non-human primate visual cortex (area V1) is a direct product of visual sensory mechanisms, and it helps explain the role of microsaccades in the maintenance of perception during steady visual fixation[15]. As there is an alteration of the visual cortex V1 architecture in amblyopia, we hypothesized that there will be a correlation between visual acuity deficit and production of microsaccades in these patients.

Several studies have investigated fixation performance in amblyopia and have shown increased fixational instability in the amblyopic eyes[16–18]. One study reported comparable fixational saccade frequency but an increase in the ocular drifts in the amblyopic eye of mild and moderate amblyopic young adults [16]. Another investigation reported comparable drift velocities in the amblyopic and the fellow eyes as well as the control subjects [18]. None of the previous studies assessed changes in the microsaccade amplitude and its correlation with the ocular drift. Such investigation might provide insight into the cause of fixation instability in amblyopia and might identify the triggers of microsaccade generation.

Here we quantitatively characterized the microsaccades (amplitude < 1 degree), fixational saccades (which includes microsaccades as well as saccades >1 degree produced during steady fixation) and drifts in the amblyopic and the fellow eye in subjects with varying severity of amblyopia and compared them to healthy controls. The drifts are known to increase with acquired vision loss[19]. Therefore, we asked whether the increase in the amplitude of the fixational saccades correlates with a pathologically increased drift.

## Methods

The experiment protocols complied with the tenets of the declaration of Helsinki, and was approved by the Cleveland Clinic institutional review board. The consent form was reviewed and approved by the Cleveland Clinic Institutional Review Board. All subjects provided written informed consent. Written informed consent was obtained from the parents or legal guardians on behalf of the minors/children enrolled in the study. The participants/legal guardians signed the consent forms. Eye movements were measured and analyzed from 11 healthy children and 36 pediatric subjects with amblyopia. We included the amblyopic subjects who had visual acuity worse than LogMAR 0.17 in the amblyopic eye, visual acuity of logMAR 0 or better in the fellow eye, and interocular difference of 2 or more logMAR lines. The amblyopes were further classified as mild (visual acuity of logMAR 0.3 or better in the amblyopic eye, n = 14), moderate (visual acuity logMAR 0.3 to 0.69, n = 15), and severe (visual acuity worse than logMAR 0.69, n = 6). The amblyopic subjects were also subdivided as having strabismic amblyopia defined as amblyopia in the presence or history of eye misalignment at distance and/or near fixation, anisometropic amblyopia when they had a difference in refractive error between the two eyes equal or greater than 1 diopter spherical equivalent or mixed mechanism if they had both strabismus and anisometropia.

### Eye movement measurements

High-resolution video-based eye tracker (EyeLink 1000®, SR Research, Ontario, Canada) was used to non-invasively measure horizontal and vertical eye positions. This method allows the identification of saccades of the amplitude of 0.1° and higher. The spatial resolution of the system is 0.01° hence allowing detection of minute differences with two decimal accuracies.

### Eye movement recordings

Each subject's head was supported on a chin-rest, 55 cm away from the LCD screen. The subjects fixated their gaze on a red visual target projected on the LCD screen on a white background in a completely dark room for 45 seconds. The target was circular; the diameter subtended 0.5° visual angle with the same contrast for all experimental sessions. To maintain the subject’s attention, we used a fixation window of 2 x 2 degree and an auditory alert (a short beep) if the subject's gaze left the area of the fixation window for >500 msec.

We used an infrared permissive filter to obtain recordings under monocular viewing conditions. Such filter allowed infrared waves to capture eye position but blocked the visible light; hence, we were able to record binocular movements during monocular viewing. Each eye was calibrated under monocular viewing conditions with best-corrected vision of the viewing eye as the subjects fixated on targets at known horizontal and vertical eccentricities. A separate calibration was performed for the two conditions (fellow eye viewing and amblyopic eye viewing conditions). The order of testing with the fellow versus the amblyopic eye was randomized. A recording trial was initiated when the calibration and its subsequent validation were acceptable with adequate accuracy for each eye. Binocular eye positions were measured at a temporal resolution of 500 Hz.

### Data analysis

We assessed eye movement traces of gaze holding for latent fixation nystagmus that was defined as a nasally directed drift followed by a refoveating temporally directed saccade. The slow phase of latent nystagmus can interfere with the analysis of drifts while the quick phase would interfere with the detection of fixational saccades. Therefore, we excluded amblyopic subjects with latent nystagmus (n = 10) from further analysis.

Eye position data were used for further analysis. We removed blinks as portions of the raw data where the pupil information was missing. In addition, we also removed portions of the data where there was an abrupt change in pupil size (>50 units/sample) as they would probably be partial blinks where the pupil is not completely occluded. We also removed 200 msec before and after each blink or partial-blink to exclude portions of the traces where the pupil could be partially occluded [20,21]. Eye position was differentiated to compute eye velocity. Fixational saccades were identified using the previously described unsupervised clustering method [22]. The technique does not require an arbitrary threshold; rather it detects fixational saccades and level of noise by automatically finding a boundary using a clustering method. This method also provides an index of reliability of the detection outcome. The reliability index is a value between 0 and 1 that is related to the signal to noise ratio in the data. Specifically it measures the distance between the distribution of saccadic events and the distributions and events extracted from noise or non-saccadic eye movements present in the data. The index correlated with the rate of detection errors of the clustering algorithm with higher error rates in recordings with smaller values of the index. Recordings with indices higher than 0.75 produced error rates less than 0.3 per second, hence providing an objective quality and reliability measure of algorithmic detection of fixational eye movements. We excluded seven amblyopic subjects and one normal control with reliability index of 0.75 or lower. Table 1 and S1 Table list the clinical and demographic features of subjects included and excluded from the analysis respectively. We separately analyzed physiologic microsaccades that were defined as the amplitude of less than or equal to 1 degree. We chose this definition as 1) most contemporary researchers by convention have used 1 degree as upper magnitude threshold as it has shown to capture more than 90% of the saccades produced during attempted fixation[23–26] 2) multiple laboratories have found that microsaccade magnitudes reached an asymptote at around 1 degree[23,27]. We also analyzed fixational saccades with larger amplitudes that could be contributing to the overall fixational instability in amblyopia.

**Table 1:** Demographic and clinical features.

	Category	Age	Acuity OD	Acuity OS	Stereopsis	Refraction OD	Refraction OS	Strabismus Near	Strabismus Distance
1	mild aniso	10	0	0.30	100 sec	+0.25	+5.75	ortho	ortho
2	mild strab	9	0	0.30	200 sec	+5.75	+6.50	8 E(T)	4 E(T)
3	mild mixed	7	0	0.17	800 sec	-1.50	-8.0	12 X(T)	20 X(T)
4	mild aniso	7	0.17	0	80 sec	+5.5	+2.5	ortho	ortho
5	mild aniso	12	-0.12	0.30	800 sec	-0.50	+1.75	ortho	ortho
6	mild mixed	8	0.30	0	100 sec	+4.0	+1.25	4 E(T)	ortho
7	mod strab	9	0	0.54	140 sec	+3.0	+3.5	8 E(T)	ortho
8	mod strab	5	0.30	-0.12	400 sec	+7.0	+7.0	6 E(T)	flick E(T)
9	mod aniso	8	0.54	0	200 sec	+8.0	+1.5	ortho	ortho
10	mod aniso	10	0.47	0	80 sec	+5.5	+3.25	ortho	ortho
11	mod aniso	4	0.39	0	100 sec	+4.25	+1.5	ortho	ortho
12	mod mixed	6	0	0.47	100 sec	+4.5	+5.75	8 E(T)	ortho
13	mod mixed	14	0.39	0	nil	+3.75	+1.25	50 ET	45 ET
14	mod mixed	7	0.47	0	400 sec	+5.25	+1.0	4–6 E(T)	4 E(T)
15	mod aniso	5	0	0.47	400 sec	plano	+2.5	ortho	ortho
16	severe strab	7	0.87	0	nil	+2.25	+2.75	55 ET	45 ET
17	severe aniso	12	0	1.3	nil	+1.25	+7.25	ortho	ortho
18	severe aniso	7	1.20	0	nil	+6.25	plano	ortho	ortho
19	severe mixed	15	-0.12	0.79	400 sec	+0.5	+3.25	20 X(T)	25 X(T)
20	control	10	0	0	40 sec	+0.25	+0.25	ortho	ortho
21	control	9	0	0	40 sec	+0.50	+0.50	ortho	ortho
22	control	7	0	0	40 sec	-1.5	-1.5	ortho	ortho
23	control	7	0	0	40 sec	+1.00	+1.00	ortho	ortho
24	control	12	-0.12	-0.12	40 sec	-3.0	-3.0	ortho	ortho
25	control	8	0	0	40 sec	+1.0	+1.25	ortho	ortho
26	control	9	0	0	40 sec	+0.25	+0.25	ortho	ortho
27	control	5	0	0	60 sec	+1.50	+1.50	ortho	ortho
28	control	8	0	0	40 sec	+1.0	+1.0	ortho	ortho
29	control	10	-0.12	-0.12	40 sec	-2.0	-2.0	ortho	ortho

Fixational saccade frequency was computed as a number of events in one second. Small rapid eye movement in the opposite direction called dynamic overshoot follows some saccades. We identified the dynamic overshoot by their very short latency (< 20 msec) between the two movements and were not considered as a “new” saccade. Thus, saccade amplitude was defined as the absolute difference between the eye positions at the start and the end of the saccade (which comprised the dynamic overshoot). The composite vectorial amplitude of horizontal and vertical displacements were determined and used for further analysis. We separately analyzed frequencies of fixational saccades of all amplitudes and microsaccades.

Drift periods were defined as epochs between fixational saccades and blinks. We removed 20 milliseconds from the start and end of each of these epochs to exclude periods of acceleration and deceleration of the eye during fixational saccades and blinks. The composite eye velocity of the ocular drifts and the composite variability of eye position were computed using the following equation.

$$composite={\left[{horizontal}^{2}+{vertical}^{2}\right]}^{\frac{1}{2}}$$

We also quantified the fixation stability by measuring a bivariate contour ellipse (BCEA) using the following equation [28].

$$BCEA=\pi \chi 2\sigma_{x}\sigma_{y}{\left(1-\rho^{2}\right)}^{1/2}$$

In the equation Χ ^2^ is a chi-square variable with two degrees of freedom, σ _x_ σ_y_ are the standard deviation of eye position in the horizontal and vertical meridian respectively, and *p* is the product moment correlation of the two position components. A log_10_ transformation was used to normalize the resulting BCEAs. All the parameters were measured for the viewing and non-viewing eye. All the data analysis was performed using custom prepared software in Matlab™ programming language.

### Statistics

We used Matlab™ (Mathworks, Natick, MA, USA) and GraphPad Prism 6™ (La Jolla, CA, USA) for statistical analysis. A Kruskal-Wallis analysis of variance test was used to compare the frequencies of microsaccades and fixational saccades, median eye position variance of ocular drifts and BCEA across amblyopia subjects (mild, moderate and severe amblyopia) and controls. A Kruskal-Wallis analysis of variance test was used to compare the frequencies of microsaccades and fixational saccades across amblyopia subtypes (anisometropic, strabismic or mixed). The Kolmogorov-Smirnov test was used to compare the distribution of fixational saccade amplitude amongst all four groups as well as in groups in whom stereopsis was present versus absent. Mann-Whitney U test was used when fixational saccade frequency comparison was made on the same subject during fellow eye versus amblyopic eye viewing condition or comparison of fixational saccade frequency in the viewing eye versus non-viewing eye. Mann-Whitney U test was also used to compare the median drift variance in the non-viewing eye during fellow eye viewing and amblyopic eye viewing conditions. Spearman correlation was used to compute a correlation between the drift variance in the viewing and non-viewing eye and fixational saccade amplitude and the variance preceding ocular drift and its velocity.

## Results

The fixation stability in amblyopia is affected by the presence of fixational saccades, ocular drifts and latent nystagmus. We investigated the effects of drifts and fixational saccades on the visual function as a contributor to fixational instability in pediatric subjects with amblyopia. We excluded subjects with latent nystagmus. We specifically investigated whether the dynamics of fixational saccades and ocular drifts are altered in amblyopia and if there is a correlation between such alterations and severity of amblyopia.

**Fig 1:**
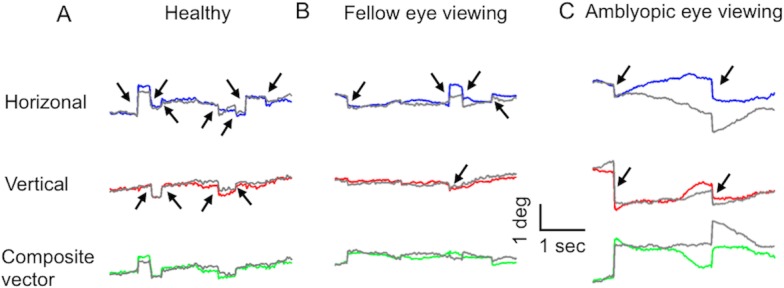
**An example of visual fixation during a 4-second epoch in a normal control (A) and a subject with severe amblyopia during fellow eye viewing condition (B) and amblyopic eye viewing condition (C).** The top two traces plots horizontal and vertical eye position along the y-axis and time along the x-axis. The bottom trace depicts the composite vector of horizontal and vertical eye positions. The gray colored trace is the non-viewing eye whereas the blue, red and green trace represents the horizontal, vertical and composite vector of the viewing eye. Short arrows represent fixational saccades.

Fig 1 depicts an example of visual fixation with best-corrected visual acuity in a normal subject (Fig 1A), in a subject with severe amblyopia in fellow eye viewing condition (Fig 1B), and in the same subject during amblyopic eye viewing condition (Fig 1C). Examples of fixational saccades (arrows) are interspersed by epochs of inter-saccadic drifts. In the illustrated example, during a 4-second epoch, the normal control subject made six fixational saccades (Fig 1A). In contrast, the subject with amblyopia made five fixational saccades during fellow eye viewing condition (Fig 1B) and two fixational saccades during amblyopic eye viewing condition (Fig 1C). The amplitude of fixational saccades and the variance in the eye position between adjacent fixational saccades was also increased in the subject with amblyopia. These observations were consistently noted in all subjects with amblyopia. We quantify these results in the subsequent sections.

### Amplitude of fixational saccades in amblyopia

**Fig 2:**
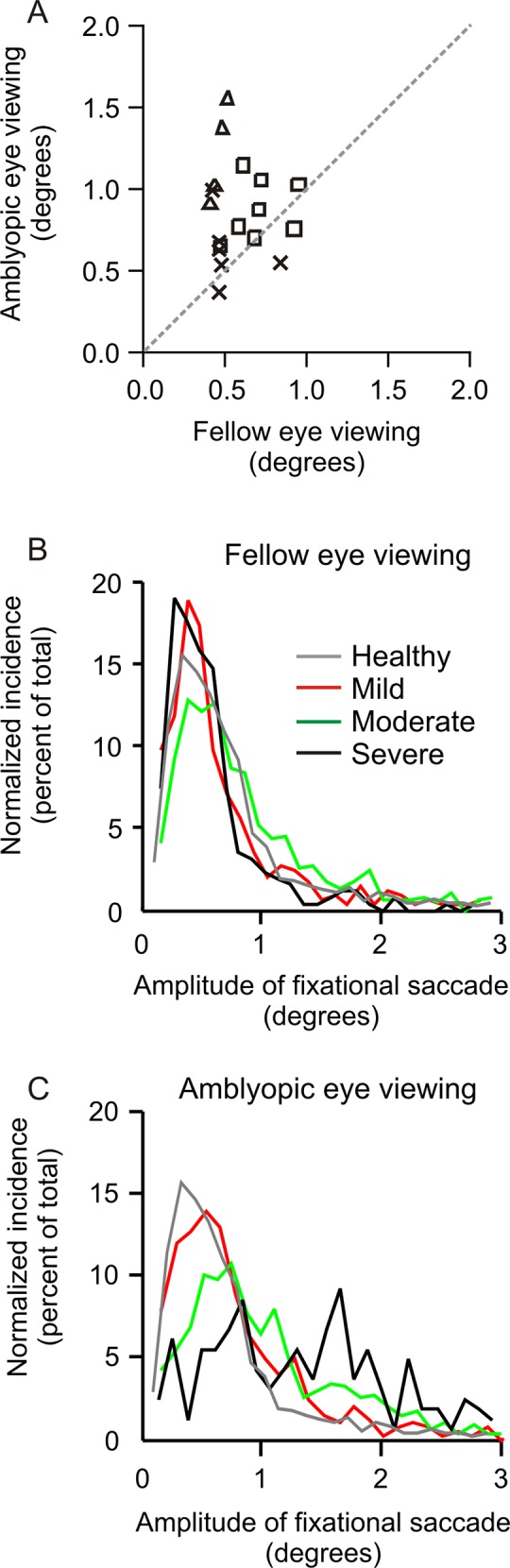
(A) Scatter plots of summary of the effects of severity of amblyopia on median amplitude of fixational saccades during fellow eye viewing condition (x-axis) and amblyopic eye viewing condition (y-axis). Each symbol depicts one subject whereas the symbol type represents severity of amblyopia. The dashed line is the equality line. (B, C) Summarizes the distribution of the fixational saccade amplitude during fellow eye viewing condition (B) and amblyopic eye viewing condition (C). A normalized number of events in a given bin is plotted along the y-axis while the x-axis represents the bins of fixational saccade amplitude of the viewing eye. The gray line illustrates the normalized frequency histogram of microsaccade amplitude in normal controls. The red, green and black lines represent mild, moderate and severe amblyopia subjects.

Fig 2A summarizes the comparison of the median amplitude of fixational saccades in amblyopic subjects during fellow eye viewing versus amblyopic eye viewing conditions. Two subjects with mild amblyopia (cross symbols) and one subject with moderate amblyopia (square symbol) had greater median fixational saccade amplitude during fellow eye viewing condition compared to amblyopic eye viewing condition. The remaining mild and moderate amblyopes and all severe (triangle symbol) were above the equality line suggesting that the median amplitude of fixational saccade in these subjects was greater when the subjects viewed with the amblyopic eye.

Fig 2B summarizes the distribution of the fixational saccade amplitude during fellow eye viewing condition. The gray line illustrates the normalized frequency of distribution of fixational saccade amplitude in controls. The population median for controls was 0.56°; it was 0.50° for mild amblyopia, 0.67° for moderate, and 0.46° severe amblyopia. It is noteworthy that the distributions overlaid upon the healthy subjects and there was no significant difference between control subjects and amblyopes (K-S statistics; p = 0.2) during fellow eye viewing condition. Fig 2C illustrates the distribution of fixational saccade amplitude during amblyopic eye viewing condition. The population median for mild amblyopia was 0.65°, it was 0.85° in moderate, and 1.28° in severe amblyopia. The distributions were significantly different in mild, moderate and severe amblyopia compared to the controls (K-S statistics; p <0.0001). This analysis suggested that the amplitude of fixational saccade is significantly larger in subjects with amblyopia. The increase is related to the severity of amblyopia, and the difference is robust during amblyopic eye viewing conditions in severe amblyopia. The interpretation of these results is that in subjects with amblyopia, during amblyopic eye viewing condition, a substantial portion of the fixational saccades do not follow the conventional definition of “microsaccades” that, if physiological, should be less than 1 degree amplitude. We then compared the distribution of amplitude in the viewing and non-viewing eye during fellow eye and amblyopic eye viewing conditions in mild, moderate and severe amblyopia subjects. We found a similar distribution in the viewing and non-viewing eye during both fellow and amblyopic eye viewing conditions in mild, moderate and severe amblyopia subjects (Table 2). We found a similar increase in fixational saccade amplitude of viewing and non-viewing eye during amblyopic eye viewing condition of subjects who did not have any stereopsis compared to those who had gross stereopsis (Table 3).

**Table 2:** Comparison of fixational saccade amplitude in viewing and non-viewing eye.

	Mild amblyopia	Moderate Amblyopia	Severe Amblyopia
Median amplitude of viewing eye (°)	0.50 ± 0.51	0.67 ± 0.57	0.46 ± 0.41
Median amplitude of non-viewing eye (°)	0.53 ± 0.55	0.71 ± 0.61	0.46 ± 0.53
K-S statistic	p = 0.16	p = 0.16	p = 0.32
Amblyopic eye viewing condition			
	Mild amblyopia	Moderate Amblyopia	Severe Amblyopia
Median amplitude of viewing eye (°)	0.65 ± 0.55	0.85 ± 0.61	1.28 ± 0.70
Median amplitude of non-viewing eye (°)	0.60 ± 0.50	0.93 ± 0.62	1.04 ± 0.77
K-S statistic	p = 0.61	p = 0.13	p = 0.13

**Table 3:** Comparison of fixational saccade amplitude in viewing and non-viewing eye.

Fellow eye viewing condition			
	Stereopsis present	Stereopsis absent	K-S statistic
Median amplitude of viewing eye (°)	0.75 ± 0.53	0.68 ± 0.58	p = 0.0014
Median amplitude of non-viewing eye (°)	0.79 ± 0.57	0.74 ± 0.63	p < 0.00001
Amblyopic eye viewing condition			
	Stereopsis present	Stereopsis absent	K-S statistic
Median amplitude of viewing eye (°)	0.92 ± 0.59	1.31 ± 0.70	p < 0.00001
Median amplitude of non-viewing eye (°)	0.90 ± 0.59	1.43 ± 0.72	p < 0.00001

### Frequency of fixational saccades in amblyopia

**Fig 3:**
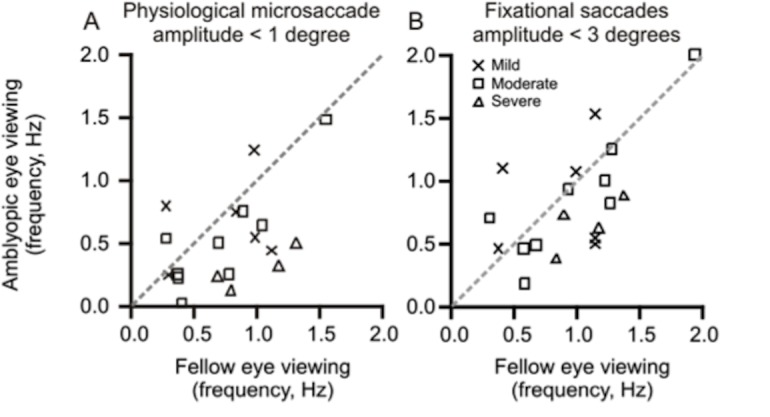
**Scatter plots of summary of the effects of severity of amblyopia on median frequency of fixational saccades less than 1° (physiological microsaccades) (A) and fixational saccades of all amplitudes (B) during fellow eye viewing condition (x-axis) and amblyopic eye viewing condition (y-axis).** Each symbol depicts one subject whereas the symbol type represents severity of amblyopia (cross symbol = mild amblyopia; square symbol = moderate amblyopia and triangle symbol = severe amblyopia. The dashed line is the equality line.

In the subsequent analysis, we assessed the frequency of microsaccades (the fixational saccades in the physiological range, < 1°) in subjects with amblyopia. Fig 3A depicts the summary of microsaccade frequency from all amblyopic subjects. The microsaccades had significantly reduced frequency in amblyopic subjects and were dependent on the viewing eye condition. There was a marked reduction in median frequency during amblyopic eye viewing condition across all four groups. It was 0.64 ± 0.34 Hz in mild amblyopia; 0.50 ± 0.42 Hz in moderate cases, and it was 0.26 ± 0.15 Hz in severe amblyopia (Kruskal-Wallis analysis of variance, chi-square value = 9.6, the total number of subjects: 29 p = 0.02). The frequencies of microsaccades during fellow eye viewing condition were comparable across all four groups. In control subjects, it was 0.83 ± 0.3 Hz; it was 0.9 ± 0.36 Hz in mild amblyopia; 0.69 ± 0.41 Hz in moderate disease, while 0.98 ± 0.30 Hz in severe amblyopia (Kruskal-Wallis analysis of variance, chi-square value = 2.3, the total number of subjects: 29, p = 0.49).

Fig 3A depict the comparison of the frequency of microsaccades in the same subject when they were viewing with the fellow eye versus the amblyopic eye. The dashed line is the equality line, and each symbol depicts one subject whereas the symbol type illustrates the severity of amblyopia. It is noteworthy that only two subjects with mild amblyopia (cross symbols) and one with moderate amblyopia (square symbols) had lower frequency during fellow eye viewing condition compared to amblyopic eye viewing condition. All other amblyopia subjects had greater median frequency during fellow eye viewing condition compared to amblyopic eye viewing condition (Mann Whitney test, p = 0.03).

We then measured the median frequency of any fixational saccade (including physiological microsaccade) in all four groups. The median frequency during fellow eye viewing condition in control subjects was 0.97 ± 0.34 Hz; it was 1.0 ± 0.37 Hz in mild amblyopia; 0.93 ± 0.50 Hz in moderate disease, while 1.0 ± 0.12 Hz in severe amblyopia (Kruskal-Wallis analysis of variance test, chi-square value = 1.1, total number of values: 29, p = 0.77). The median frequency during amblyopic eye viewing condition in control subjects was 0.97 ± 0.33 Hz; it was 0.80 ± 0.43 Hz in mild amblyopia; 0.82 ± 0.53 Hz in moderate disease, while 0.6 ± 0.22 Hz in severe amblyopia (Kruskal-Wallis analysis of variance test, chi-square value = 3.5, total number of values: 29, p = 0.3). The median frequencies of fixational saccades of all amplitude in all amblyopic subjects were comparable during fellow eye viewing and amblyopic eye viewing conditions (Mann-Whitney p = 0.2) (Fig 3B). We found a similar microsaccade and fixational saccade frequencies in all three categories of amblyopia subtypes (anisometropia, strabismic or mixed) during fellow eye viewing and amblyopic eye viewing conditions (Table 4).

**Table 4:** Frequency of microsaccades and fixational saccades.

Fellow Eye Viewing Condition				
	Anisometropic	Strabismic	Mixed	Kruskal Wallis analysis of variance
Median microsaccade frequency (Hz)	0.77 ± 0.38	0.61 ± 0.47	0.93 ± 0.36	p = 0.82
Median Fixational saccade frequency(Hz)	0.93 ± 0.47	0.78 ± 0.46	1.14 ± 0.29	p = 0.85
Amblyopic Eye Viewing Condition				
	Anisometropic	Strabismic	Mixed	
Median microsaccade frequency (Hz)	0.50 ± 0.42	0.51 ± 0.30	0.37 ± 0.39	p = 0.97
Median fixational saccade frequency(Hz)	0.82 ± 0.48	0.78± 0.38	0.55 ± 0.46	p = 0.92

We compared the frequency of the microsaccades in the non-viewing eye to the viewing eye during fellow eye viewing and amblyopic eye viewing conditions. The frequencies in the non-viewing eye were comparable to the viewing eye during both fellow eye and amblyopic eye viewing conditions (p = 0.9 and p = 0.7 respectively, Mann-Whitney test). We also compared the frequency of the fixational saccade in the non-viewing eye to the viewing eye during fellow eye viewing and amblyopic eye viewing conditions. The frequencies were similar during both fellow eye and amblyopic eye viewing conditions (p = 0.8 and p = 0.7 respectively, Mann-Whitney test).

### Main- sequence relationship of fixational saccades in amblyopia

**Fig 4:**
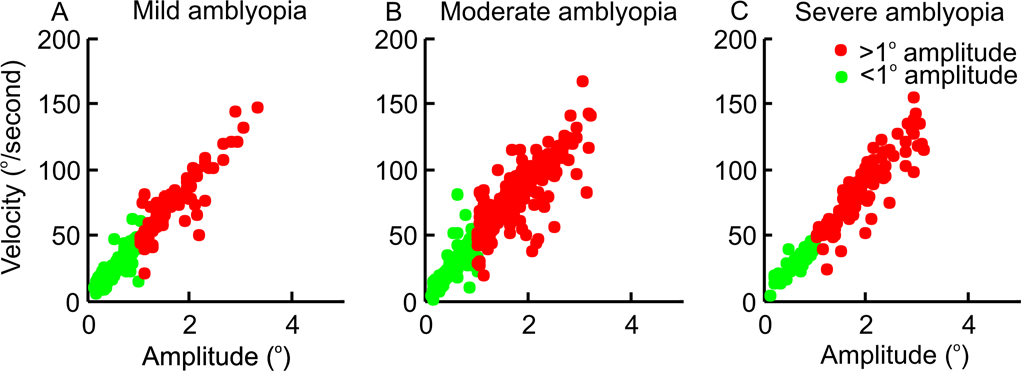
Comparison of amplitude to velocity relationship of physiological fixational saccades less than 1 degree amplitude (microsaccades, green symbols) and those with pathologically increased amplitudes in amblyopia (red symbols). Both symbols fall on the same continuum suggesting a common neural mechanism generating both types of fixational eye movements. Summary of all fixational saccades from all subjects are illustrated. Panel A depicts mild amblyopia; B is moderate; and C is severe amblyopia.

We asked whether abnormally increased amplitude of fixational eye movements follows physiological relationship between the saccade amplitude and velocity. Therefore, we compared the main-sequence relationship for microsaccades (<1° amplitude) and fixational saccades with abnormally increased amplitude (>1°). Fig 4 depicts such comparison for mild, moderate and severe amblyopia. It was clear that all fixational eye movements followed the continuum.

### Inter-saccadic ocular drift in amblyopia

**Fig 5:**
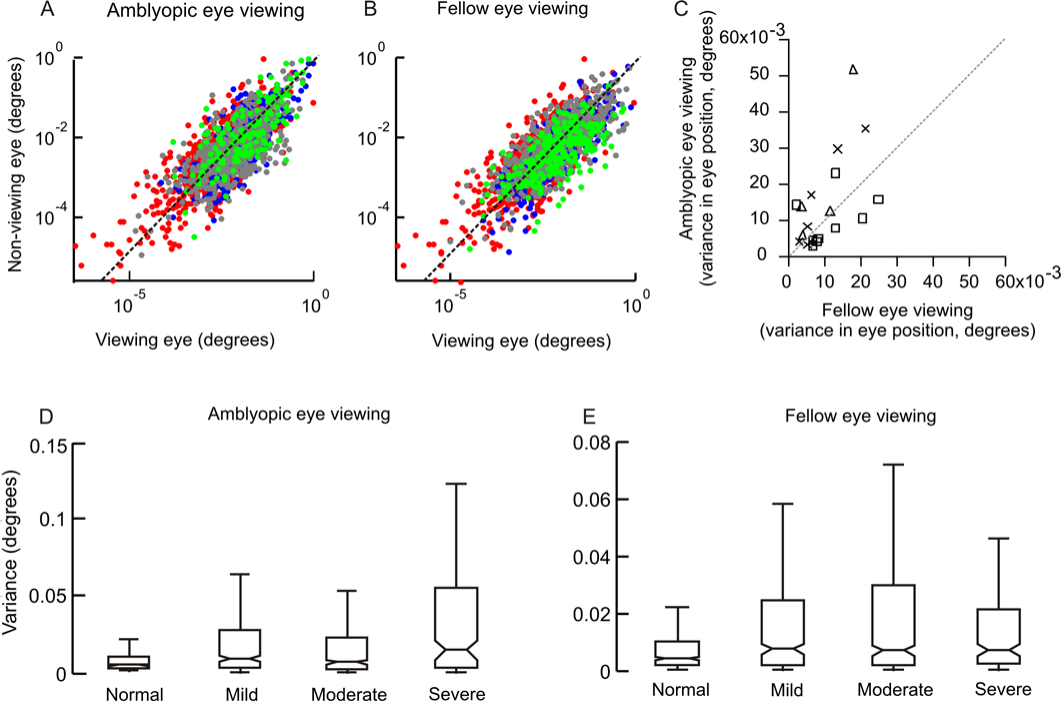
The variance of composite eye position of the viewing and non-viewing eye during fellow eye (A) and amblyopic eye viewing (B) conditions. In both cases, each symbol is one drift (epoch of eye position between two consecutive fixational saccade). Each color represents the severity of amblyopia (gray: mild; blue: moderate; red: severe). The dashed line is the equality line. Panel C plots the median variance of the non-viewing eye during fellow eye viewing and amblyopic eye viewing conditions. Each symbol represents a subject where the symbol type indicates the severity of amblyopia. Box and whisker plots of the effects of severity of amblyopia compared to normal controls on the composite variance in eye position during fellow eye viewing condition (D) and amblyopic eye viewing condition (E). The length of each box depicts the interquartile interval; notch suggests 95% confidence interval around the median (if the two notches of a box-whisker plot do not overlap it offers evidence of a statistically significant difference between the medians) and the horizontal line in the center of the notch is the median value. Overlapping notches indicate a lack of difference in the median values of two populations.

Increased eye velocity and variance of eye positions during drifts (epochs between two consecutive fixational saccades) are a well-known consequence of visual loss[19]. We analyzed the drifts in amblyopic subjects during fellow and amblyopic eye viewing conditions. Fig 5 depicts the variance of composite eye position of the viewing and non-viewing eye during fellow (Fig 5A) and amblyopic eye viewing (Fig 5B) conditions. In both cases, each symbol is one drift. Each color represents the severity of amblyopia. The dashed line is the equality line. All data points are on both sides of the equality line. There was a strong correlation between drift variance of the viewing and non-viewing eye during both fellow eye viewing condition (mild amblyopia: r = 0.82, p<0.0001; mod amblyopia: r = 0.76, p<0.0001; severe amblyopia: r = 0.69,p <0.0001) and amblyopic eye viewing condition (mild amblyopia: r = 0.76, p<0.0001; mod amblyopia: r = 0.69, p<0.0001; severe amblyopia: r = 0.74,p <0.0001). We then measured the median variance of the non-viewing eye during fellow eye viewing and amblyopic eye viewing conditions (Fig 5C). Each symbol represents a subject where the symbol type indicates the severity of amblyopia. There was no difference in variance of the non-viewing eye during fellow eye viewing versus amblyopic eye viewing conditions in all 3 groups (mild amblyopia: p = 0.67, mod amblyopia: p = 0.97; severe amblyopia: p = 0.11; Mann Whitney U test). These results suggest comparable variance in the eye position, regardless of the viewing condition. These results contrast with fixational saccades, whose amplitude is further increased, and the frequency is reduced during amblyopic eye viewing condition compared to the fellow eye viewing condition. This finding supports the lack of dependence of fixational saccades on the amplitude and velocity of drifts.

Patients with vision loss tend to have greater drifts in vertical eye position compared to horizontal eye position. We, therefore, assessed whether the variance in eye position changes with the severity of amblyopia. During both amblyopic and fellow eye viewing condition the variance was higher in amblyopes compared to healthy subjects (Fig 5D and 5E). The variance was highest during amblyopic eye viewing condition in severe amblyopia (median composite eye position variance for control subjects: 0.003°; mild amblyopia: 0.007°; mod amblyopia: 0.005°; severe amblyopia: 0.012°). Table 5 depicts the analysis for horizontal and vertical eye position variance done separately in all three amblyopia groups their comparison with controls.

**Table 5:** Characteristics of ocular drifts.

Fellow eye viewing condition					
	Healthy controls	Mild amblyopia-	Moderate Amblyopia	Severe Amblyopia	(Kruskal Wallis analysis of variance)
Horizontal eye position variance (°)	0.001 ± 0.01	0.002 ± 0.02	0.002 ± 0.02	0.002 ± 0.01	p <0.0001
Vertical eye position variance (°)	0.003 ± 0.02	0.004 ± 0.13	0.004 ± 0.04	0.003 ± 0.01	p < 0.0001
Amblyopic eye viewing condition					
	Healthy controls	Mild amblyopia-	Moderate Amblyopia	Severe Amblyopia	
Horizontal eye position variance (°)	0.001 ± 0.01	0.003 ± 0.5	0.002 ± 0.8	0.006 ± 0.1	p <0.0001
Vertical eye position variance (°)	0.003 ± 0.02	0.003 ± 0.15	0.002 ± 0.07	0.006 ± 0.12	p <0.0001

### Measurement of BCEA in amblyopia

We measured the BCEA of the viewing and non-vieiwng eye during fellow eye-viewing and amblyopic eye-viewing conditions in all four groups. The best fixation stability was evident in healthy controls, the fixation instability increased with increases in the severity of amblyopia. The fixation instability was more pronounced in both the viewing and non-viewing eye during amblyopic eye viewing condition (Table 6).

**Table 6:** Bivariate contour ellipse analysis.

Fellow eye viewing condition					
log_10_ [BCEA(deg^2^)]	Healthy controls	Mild amblyopia	Moderate amblyopia	Severe amblyopia	Kruskal Wallis analysis of variance
Viewing eye	-0.48 ± 0.48	-0.05 ± 0.34	0.28 ± 0.75	0.32 ± 0.32	p = 0.02
Non-viewing eye	-0.44 ± 0.43	0.17 ± 0.40	0.34 ± 0.58	0.35 ± 0.31	p = 0.004
Amblyopic eye viewing condition					
	Healthy controls	Mild amblyopia	Moderate amblyopia	Severe amblyopia	
Viewing eye	-0.48 ± 0.48	0.07 ± 0.31	0.35 ± 0.61	0.73 ± 0.83	p = 0.003
Non-viewing eye	-0.44± 0.43	0.16 ± 0.26	0.61 ± 0.49	0.85 ± 0.62	p = 0.004

### Correlation analysis between fixational saccade and intersaccadic drift

We did not find a correlation between the amplitude of fixational saccades and the eye position variance or the peak eye velocity of the preceding drift in all three groups of amblyopia subjects (Table 7). In other words, the digression of the eyes due to the drifts away from the visual target does not determine the amplitude of the subsequent fixational saccade.

**Table 7:** Correlation of fixational saccade amplitude with peak drift velocity and eye position variance.

	Fellow eye viewing (regression coefficient)			Amblyopic eye viewing (regression coefficient)		
	Mild	Moderate	Severe	Mild	Moderate	Severe
Velocity	0.06	0.02	0.004	0.05	0.05	0.008
Position	0.0005	0.0007	0.007	<0.0001	0.002	0.004

## Discussion

The three main findings of our study are as follows. (1) The subjects with amblyopia have a significant decrease in microsaccade (the physiological fixational saccade) frequency. (2) There is an increase in the amplitude of fixational saccades during amblyopic eye viewing condition. (3) The variance and velocity of ocular drifts are increased during both fellow eye and amblyopic eye viewing conditions. The changes in fixational saccade production correlated with the severity of amblyopia, but the increases in fixational saccade amplitude did not correlate with the drift variance or velocity. We also found impairment in the fixational eye movements even when the fellow eye viewed the target. Our key interpretation is that abnormal increase in the amplitude of fixational saccades and reduction in their frequency in the amblyopic eye is not a compensatory phenomenon resulting from an increased drift velocity and variance due to visual compromise.

Our results are distinct compared to prior studies that have described fixation stability in amblyopia. One study evaluating 13 subjects with mild to moderate amblyopia found similar fixation stability of the fellow eye compared to that of the control subjects, but the amblyopic eye had significantly lower fixation stability[16]. There was no direct correlation between the severity of amblyopia and fixation stability of the amblyopic eye, however, the interocular difference in visual acuity (acuity deficit) correlated significantly with the fixation stability of the fellow eye during monocular and binocular viewing conditions [16]. Furthermore, there were no differences in the fixational saccade frequency in amblyopes compared to controls leading to a conclusion that increased fixation instability in amblyopia is due to slow ocular drifts alone. There are several reasons for the disparity in their study and our results. Unlike the circular red target used in our study, the previous study has used a three-degree red cross to study fixational abnormalities. Type of fixation stimulus affects both, the microsaccades and the drifts [29]. It is possible that the type of fixation stimulus (circular versus cross hair target) may have a different effect on microsaccades and drift production in amblyopic eyes accounting for the difference in results seen in the current study, although, frequency of microsaccades should not be affected [29]. Also, the inclusion of a limited number of subjects with mild to moderate amblyopia, but lack of severe amblyopic subjects in the cohort, could also account for the absence of difference in the fixational saccade frequency.

Another study evaluated fixation instability in a large cohort of amblyopic subjects with varying severity using a Nidek microperimeter and bivariate contour ellipse measures of the fixation points. This technique objectively assesses the fixation instability but cannot characterize the various subtypes of eye movements leading to such instability[17]. There was an increase in fixation instability in the amblyopic eye compared to the fellow eye or the healthy subjects, but the study did not reveal whether it is a consequence of increased drifts or fixational saccades. Bivariate contour ellipse analysis in the amblyopic eyes of our subjects showed a similar increase in fixation instability.

A recent study assessed fixational saccades with scanning laser ophthalmoscope reported an increased saccade frequency with amplitude of less than 1 degree in amblyopic subjects [18]. There are several explanations for such a discrepancy. The authors chose up to 40 frames of out of 900 video frames and initial 10 seconds of each video frame thus introducing a bias due to truncated sample size. Also, the reported range of fixational saccade frequency in control subjects was two times higher (0.6–5 Hz) than that reported in the literature (1–2 Hz). The authors speculate that possible subtle head motion during their experiments could have resulted in a higher frequency. We used high-resolution video oculography and secure head stabilization to collect data. This technology allows identification of saccades that are as small as 0.1° and spatial resolution of 0.01°. A key difference is the temporal resolution of the eye movements recording machine, which varies from 25 Hz for methods such as scanning laser ophthalmoscope and Nidek microperimeter to 1000 Hz for the modern eye-trackers. Low temporal resolution machines like scanning laser ophthalmoscope or Nidek microperimeter pose difficulties in accurately parsing the eye position data between fixations and saccades [30]. Such spatio-temporal resolution disparity could account for the differences seen in our results compared to previous studies.

We recognize the challenges of obtaining reliable data that measures small magnitude of fixational eye movements and its critical dependence on the sensitivity and precision of eye tracking systems. However, multiple studies in normal controls and patients have shown significant agreement (95%) between the video tracker utilized in the current study and the scleral search coils (the gold standard in eye movement recordings)[31,32]. In fact, the availability of these commercial video-trackers with excellent precision and accuracy with ease of use has renewed an interest of ocular motor scientists in studying fixational eye movements. In addition, our subjects were children; use of scleral search coil that requires wearing a coil embedded in contact lens was deemed difficult. Thus, we opted to use the Eyelink video-tracker, which has been successfully used by multiple investigators in different laboratories to measure fixational eye movements in normal controls[8,10,33] and in disease states[32,34].

Our experiment also addresses some critical issues for fixational saccade detection. We used an unsupervised method to detect fixational saccades using clustering techniques in varying severity of amblyopic subjects. The advantages of this method are that it provides an index of reliability related to the signal-to-noise ratio, a critical feature when measurements are done in the pediatric age group. Finally, our analysis strategy does not rely on the binocular criterion for fixational saccade detection, a useful feature in case of subjects with strabismus and amblyopia where the disconjugacy between the two eyes is very likely. In addition, exclusion of patients with latent nystagmus from our cohort limited the generalizability of our results to all amblyopic children.

We used longer fixation times (45 seconds) compared to most studies in the literature. While we recognize that prolonged fixation could potentially affect the gaze stability; previous studies have used 45 seconds trials to study fixational eye movements obtaining results that are comparable to investigations with shorter trials [35]. A more recent experiment used 60 seconds fixation trials to show the events categorized as microsaccades in data recorded with scleral search coil matched those in data recorded with a video method 95% of the time [31]. Our choice of longer fixation times was to allow optimization of microsaccade detection, increase the sample size of the drift and microsaccades, and reliably identify the correlation between ocular drifts and microsaccade production. Qualitatively the subjects did not consider the testing time to be too long. Furthermore, to maintain the subjects’ attention we used a fixation window of 2 x 2 degree and an auditory alert (a short beep) if the gaze left the area of the fixation window for more than 500 ms.

Fixational saccades (elicited during fixation task) and exploratory saccades (elicited during free scene viewing condition) are considered as an ocular motor continuum. Contrary to the prior belief that microsaccades are absent during free viewing or visual exploration, some of the recent studies have shown that microsaccades are produced during visual exploration and could serve as a sampling strategy to capture fine details in the visual scene. It would be difficult to identify fixational saccades from exploratory saccades during free viewing condition. Thus, by having subjects perform a prolonged fixational task, we could identify characteristics of fixational saccades and use these parameters to study fixational saccades produced during visual exploration. Our future studies will apply the findings of this simple experiment protocol consisting of the prolonged fixation to understand the effects of amblyopia in real world visual exploration tasks.

To date, the literature has shown increased fixation instability in amblyopia with varying roles of drifts and fixational saccades contributing to it. Whether such fixation instability is a contributor to vision loss or is a consequence of vision loss is not clear. It is well known that the performance of different classes of eye movements is optimized by visual feedback[36]. The neural integrator plays a crucial role in gaze stability as it integrates the premotor velocity signals into eye position signal[36]. Recent studies have shown that the neural integrators specify the position of each eye, hence maintaining the gaze stability in each eye. The integrator accomplishes this by receiving visual information from each eye. Diminished visual feedback results in gaze instability due to loss of inputs that normally optimize the performance of the neural network (integrator) for eye movements. Such breakdown of the neural integrator network could account for the gaze instability in non-amblyopic subjects with vision loss[19]. Also, the resultant gaze instability affects both eyes but is mainly seen in the eye with poor vision. The data from our study showed increased horizontal eye drifts in both fellow and amblyopic eye compared to controls. This supports the hypothesis that loss of binocular cues due to monocular impairment of vision affects gaze stability in both eyes but mainly in the amblyopic eye.

Data from the current study as well as previous literature in healthy subjects with uncorrected refractive error and during complete darkness supports that altered frequency and amplitudes of fixational saccades are a consequence of visual acuity loss in amblyopic patients. In addition, our data shows that fixational eye movement abnormalities (abnormal ocular drifts) are also seen in the fellow eye of the amblyopic patients. Furthermore, we show that the severity of increase in the amplitude of fixational saccades does not correlate with the variance in eye position and eye velocity during drifts. Hence, changes in the kinematic properties of fixational saccades are not the consequence of increased eye position variance and eye velocity due to visual deprivation; instead, it could be due to the visual acuity deficit in amblyopia. Finally, ours and other studies have shown that the fellow eye in amblyopia also has fixational abnormalities. These findings suggest that amblyopia is not a monocular problem but it should be considered a binocular disorder that merits a reformulation of the current monocular treatment approach.

## Supporting Information

S1 Dataset Exemplary dataset. (XLSX)

S1 Table The table depicts clinical and demographic summary of subjects who were excluded from the analysis, either due to the presence of latent nystagmus or low reliability index. (DOCX)
