# Supplementary material for: Abnormal Fixational Eye Movements in Amblyopia
Source: PLoS One. 2016 Mar 1;11(3):e0149953. doi: 10.1371/journal.pone.0149953 (PMC4773232; doi:10.1371/journal.pone.0149953)
Supplement: S1 Table — (DOCX) [file pone.0149953.s002.docx]

| Supplemental Table 1 Clinical and demographic features of patients excluded from analysis | | | | | | | | | |
| --- | --- | --- | --- | --- | --- | --- | --- | --- | --- |
|  | Category | Age | Acuity  OD | Acuity  OS | Stereopsis | Refraction  OD | Refraction  OS | Strabismus  Near | Strabismus Distance |
| 30 | Control  (LR)* | 9 | 0 | 0 | 40 sec | +1.0 | +0.75 | ortho | Ortho, |
| 31 | Mild aniso (LR)* | 14 | 0.30 | 0 | nil | ‘-7.0 | ‘-1.0 | 6-8 X(T) | 12-14 X(T) |
| 32 | Mild mixed (LR)* | 8 | 0.30 | 0 | 400 sec | ‘-11.5 | ‘-6.25 | 10 X(T) | 16-18 X(T) |
| 33 | Mild mixed (LN)** | 10 | 0.17 | 0 | nil | ‘+5.75 | ‘+3.75 | 2-4 E(T) | Flick E(T) |
| 34 | Mild aniso  (LR)* | 6 | 0 | 0.17 | 140 sec | ‘+0.25 | ‘+2.75 | ortho | ortho |
| 35 | Mild mixed (LR)* | 9 | 0 | 0.30 | nil | ‘+4.25 | ‘+5.5 | 6-8 E(T) | ortho |
| 36 | Mild mixed (LN)** | 17 | 0.30 | 0 | nil | ‘-4.25 | ‘-2.0 | 45 X(T) | 45 X(T) |
| 37 | Mild mixed (LN)** | 5 | 0.30 | 0 | 800 sec | ‘+4.25 | ‘+2.0 | 20 X(T) | 25 X(T) |
| 38 | Mild strab (LN)** | 15 | 0 | 0.17 | nil | ‘+1.0 | ‘+1.0 | 20 XT | 20 XT |
| 39 | Moderate aniso (LR)* | 8 | -0.12 | 0.60 | 140 sec | plano | ‘-11.5 | ortho | ortho |
| 40 | Moderate  Mixed (LR)* | 8 | 0 | 0.39 | nil | ‘-4.0 | ‘-9.0 | 14 X(T) | 18 X(T) |
| 41 | Moderate strab (LN)** | 6 | 0 | 0.39 | nil | ‘+6.25 | ‘+6.5 | 2-4 E(T) | ortho |
| 42 | Mod mixed (LN)** | 6 | 0 | 0.54 | 400 sec | ‘+4.5 | ‘+6.5 | 14 E(T) | 12 E(T) |
| 43 | Moderate mixed (LN)** | 15 | 0.39 | 0 | nil | ‘+2.75 | ‘+0.5 | 45 X(T) | 50 X(T) |
| 44 | Moderate  strab(LN)** | 11 | 0 | 0.60 | nil | ‘+3.25 | ‘+3.25 | 10 E(T) | 8 E(T) |
| 45 | Severe mixed (LR)* | 9 | 1.3 | 0 | nil | ‘+8.0 | ‘+1.0 | 18 E(T) | 20 E(T) |
| 46 | Severe mixed (LN)** | 12 | 1 | 0 | nil | ‘+5.5 | ‘+1.75 | 10 ET | 8 ET |
| 47 | Severe aniso (LN)** | 6 | 1 | 0 | nil | ‘+1.5 | “+6.0 | ortho | ortho |

* LR= Low reliability, ** LN = Latent nystagmus
